# The Long Non-coding Road to Atherosclerosis

**DOI:** 10.1007/s11883-020-00872-6

**Published:** 2020-08-09

**Authors:** Tatjana Josefs, Reinier A. Boon

**Affiliations:** 1 Department of Physiology, Amsterdam Cardiovascular Science, VU University, Amsterdam UMC, Postbus 7057, 1007 MB Amsterdam, The Netherlands; 2 Institute for Cardiovascular Regeneration, Centre for Molecular Medicine, Goethe University, Frankfurt am Main, Germany; 3 German Center for Cardiovascular Research (DZHK), Frankfurt am Main, Germany

**Keywords:** lncRNA, Atherosclerosis, Cardiovascular disease

## Abstract

**Purpose of Review:**

To summarize recent insights into long non-coding RNAs (lncRNAs) involved in atherosclerosis. Because atherosclerosis is the main underlying pathology of cardiovascular diseases (CVD), the world’s deadliest disease, finding novel therapeutic strategies is of high interest.

**Recent Findings:**

LncRNAs can bind to proteins, DNA, and RNA regulating disease initiation and plaque growth as well as plaque stability in different cell types such as endothelial cells (ECs), vascular smooth muscle cells (VSMCs), and macrophages. A number of lncRNAs have been implicated in cholesterol homeostasis and foam cell formation such as *LASER*, *LeXis*, and *CHROME*. Among others, *MANTIS*, *lncRNA-CCL2*, and *MALAT1* were shown to be involved in vascular inflammation. Further regulations include, but are not limited to, DNA damage response in ECs, phenotypic switch of VSMCs, and various cell death mechanisms. Interestingly, some lncRNAs are closely correlated with response to statin treatment, such as *NEXN-AS1* or *LASER*. Additionally, some lncRNAs may serve as CVD biomarkers.

**Summary:**

LncRNAs are a potential novel therapeutic target to treat CVD, but research of lncRNA in atherosclerosis is still in its infancy. With increasing knowledge of the complex and diverse regulations of lncRNAs in the heterogeneous environment of atherosclerotic plaques, lncRNAs hold promise for their clinical translation in the near future.

## Introduction

The fate of atherosclerosis is dependent on the phenotype of a variety of highly plastic cells in atherosclerotic plaques, and their myriad functions are transcriptionally and post-transcriptionally regulated in response to environmental stimuli. With advances in genomic tools, a new variable for gene regulation, namely non-coding RNA (ncRNA), has been introduced. This previously considered “evolutionary junk” makes up the majority of the transcribed human genome with only 2% being transcribed as RNA encoding proteins [1, 2]. Accumulating evidence shows that ncRNAs contribute to the regulation of networks in physiological and pathophysiological mechanisms, including those of cardiovascular diseases (CVDs).

CVDs are responsible for the majority of morbidity and mortality worldwide, and despite developments in scientific discoveries, clinical cardiology and public health leading to improved outcomes in patients who suffered CV events, its prevalence is still expected to rise [3–5]. Thus, novel strategies to diagnose, prevent, and treat CVD are desperately needed.

Early GWAS gave first insights into the importance of ncRNA in CVD, defining the most significantly associated locus with coronary artery diseases (CAD)—Chr9p21—to contain a stretch of 58 kilobases (kb) of ncRNAs [6–9]. The locus was shown in subsequent studies to associate with atherosclerosis [10–12] and different atherosclerosis endpoints such as myocardial infraction, stroke andaneurysms [8, 13–19]. Over the past decade, the number of CAD risk loci rose from originally 9 [6] to 243 in 2017 [20].

While early research in the field of ncRNA focused on principal RNA participants in gene expression, namely messenger, ribosomal, and transfer RNAs, research interest expanded to micro RNAs (miRNA) in early 2000*.* Novel genomic technologies including the availability of fast and cost-effective sequencing technologies as well as computational resources, opened up the field for long ncRNA (lncRNA) and circular RNA (circRNA) [21]. Multiple lncRNAs have been described to play a role in atherosclerosis, the main underlying pathology of CVD. Thereby, lncRNAs are implicated in several atherogenic processes, such as endothelial dysfunction, lipid deposition, and inflammation, and have been shown to be expressed in different cell types known to be present in atherosclerotic lesions (e.g., endothelial cells (ECs), vascular smooth muscle cells (VSMCs), macrophages).

LncRNAs still lack a clear classification but are generally defined as ncRNA > 200 nucleotides (nt) long and make up the largest part of ncRNA [22]. However, up to date, fewer than 5% have been characterized, partly due to poor conservation among species [23, 24]. While lncRNAs by definition have no protein coding potential, hence mostly lack functional initiation and termination codons [25], some lncRNAs have surprisingly been found to translate into micropeptides [26, 27], complicating the classification of lncRNAs further. Although the lncRNA classification is still unclear, it became apparent that this heterogeneous group of ncRNA can bind to DNA, RNA, proteins, or a combination thereof, likely due their capacity to fold into various thermodynamically stable structures [28].

This review aims to summarize recent studies on lncRNAs in the field of atherosclerosis and is divided into their binding ability with proteins, DNA, and RNA.

## Mechanisms of lncRNAs

LncRNAs are present in the nucleus and in the cytoplasm and therefore are able repress and activate genes on transcriptional and post-transcriptional levels. According to their position on the genome and adjacent genes, lncRNAs can be classified as sense, antisense, bidirectional, intronic, or intergenic lncRNAs and act in *cis* or *trans* (regulating genes in close proximity or further away, respectively). According to their functions, lncRNAs can also be classified as signaling, decoy, guide, and scaffold lncRNAs, while one lncRNA can have multiple archetypes [29].

Transcriptional regulatory mechanisms include interaction with chromatin-modifying complexes, transcriptional regulators, and DNA [30]. These interactions can either repress or activate gene expression, depending on the nature of enzymes bound to chromatin complexes and the type of function for interacting lncRNA. If transcribed in response to stimuli, so-called signaling lncRNAs serve as molecular signals and regulate gene expression via one of the mechanisms described below. Their transcription is time and location specific [31]; and thus, their presence may also reflect cell condition, state, and transcriptional activity. Decoy lncRNAs can impair the interaction of transcriptional regulators with their target genes by, for instance, mimicking DNA-binding sites, and impair downstream effector functions. Guide lncRNAs on the other hand can enhance downstream effector functions by aiding localization of transcriptional regulators to specific regions. Additionally, lncRNAs can mediate protein-protein interactions resulting in organization of nuclear subdomains, e.g., polycomb group proteins (scaffold lncRNAs). By direct RNA-DNA interaction, lncRNAs can display enhancer-like activity (enhancer RNA; eRNA) or form RNA-DNA triplex structures repressing gene expression via blocking the assembly of the pre-initiation complex. Not only the presence of lncRNAs but also its transcription can modify mRNA expression. Like mRNAs, lncRNAs are believed to be mainly transcribed by RNA polymerase II (Pol II) [25, 32] and Pol II-bound chromatin-modifying complexes can deposit histone modifications while moving along the DNA locus. Further, lncRNA transcription-dependent chromatin modifications can also affect binding affinity for regulatory factors.

Post-transcriptional regulatory mechanisms include the interference with pre-mRNA splicing and both positive and negative implications on mRNA translation/stability. For instance, antisense lncRNAs can upregulate mRNA translation through interaction with the 5′region of mRNA, while binding of Alu-element containing lncRNA to mRNA Alu-elements at the 3′UTR results in Staufen-mediated decay of the mRNA. Further, direct or indirect interaction of lncRNAs with miRNAs has regulatory effects by either masking miRNA binding sites on target mRNA or by miRNA sequestration (competitive endogenous RNA).

Finally, several recent publications show that some transcripts annotated as lncRNA actually function as mRNAs and produce small proteins [27, 33], making the determination of the exact mechanism by which a lncRNA elicits its effect complex.

## LncRNA-Protein Interactions in Atherosclerosis

**Fig. 1:**
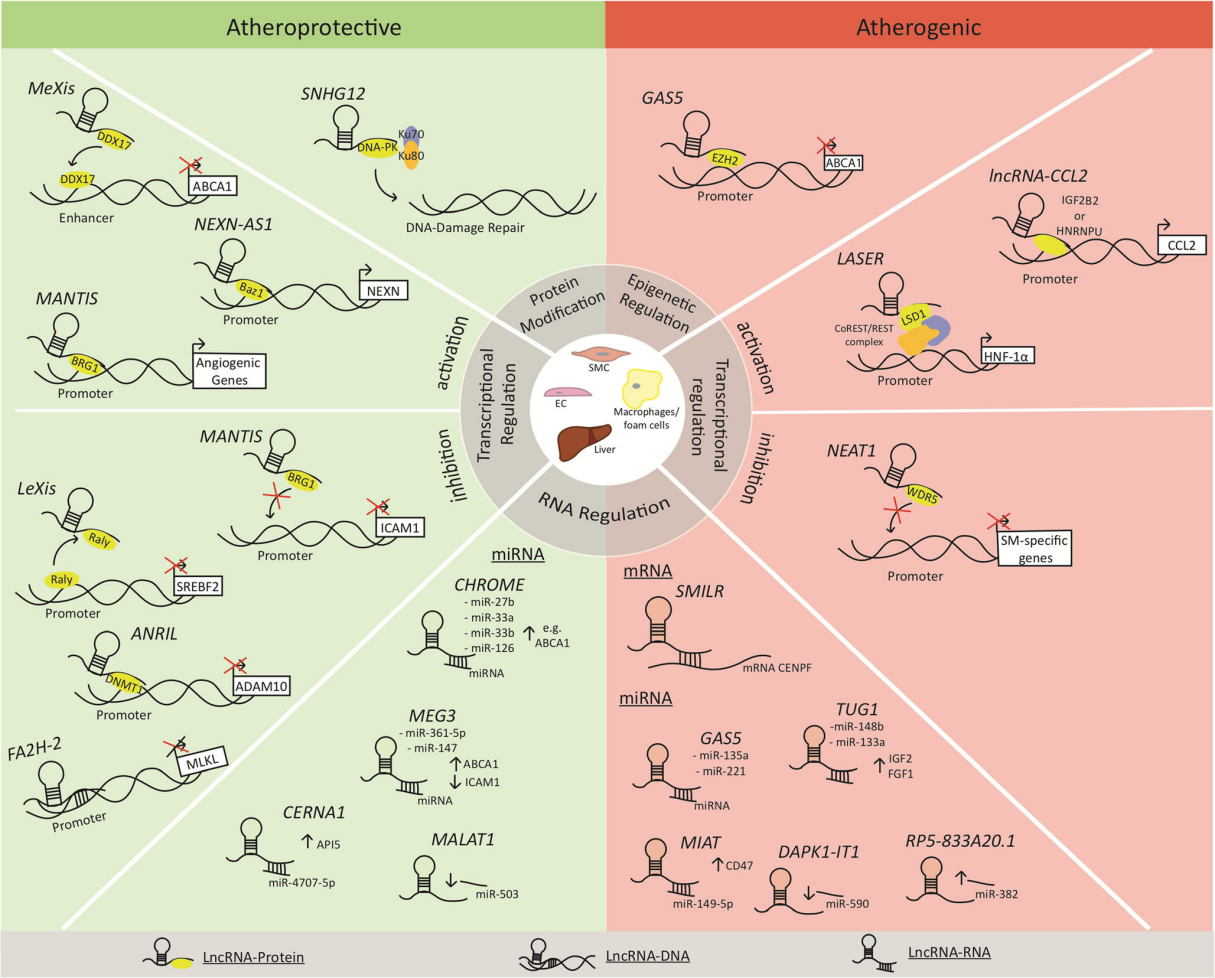
Atheroprotective and atherogenic mechanisms of lncRNAs

LncRNAs are able to regulate epigenetic changes, transcription, alternative splicing, and translation via the modulation of protein activity, localization, and structure (Fig. 1).

LncRNA *LASER* (Lipid Associated Single nucleotide polymorphism gEne Region) binds to LSD1 (lysine-specific demethylase; member of CoREST/REST complex), leading to decreased H3K4me demethylation at the promoter region of the HNF-1α gene and subsequently to increased PCSK9 (Proprotein convertase subtilisin/kexin type 9) expression in hepatocytes [34]. As PCSK9 directs low-density lipoprotein receptors (LDLR) towards degradation, *LASER* expression is positively correlated with circulating cholesterol levels (total cholesterol, LDL, apoB100) in vitro and in vivo as well as with PCSK9 in statin-free patients. Statin (=HMG-CoA reductase inhibitor) treatment lowers circulating atherogenic lipids via blocking cholesterol biosynthesis in the liver and increases *LASER* expression. Hence, its expression is regulated by a cholesterol-mediated feedback loop and represents a potential target to augment the effect of statin treatment. Also implicated in cholesterol homeostasis is *LeXis* (Liver-expressed Liver X Receptor (LXR)–induced sequence) [35], a lncRNA that binds the ribonucleoprotein RALY in hepatocytes, and inhibits its occupancy at cholesterol biosynthetic gene promoters, such as *Srebf2*. Using a liver-specific adeno-associated virus (AAV8)–based gene approach to increase *LeXis* expression in western diet (WD)–fed *Ldlr*^*−/−*^ mice was associated with a decrease in hepatic and circulating lipid levels and reduced atherosclerosis [36]. This indicates that the *LeXis*-mediated crosstalk between LXR and sterol regulatory element binding protein (SREBP) transcription factors could therapeutically be harnessed to maintain cholesterol homeostasis in CVD risk patients.

While regulating the secretion of atherogenic lipoproteins from the liver into the circulation is a central step in maintaining cholesterol homeostasis, thus preventing pathogenic deposition of these lipoproteins in the artery wall, another pivotal process is the removal of excess cholesterol from atherosclerotic plaques. Cholesterol efflux represents the first step of the reverse cholesterol transport (RCT) and is done to nascent apolipoprotein A1 via ABCA1 transporter or to mature high-density lipoprotein (HDL) via ABCG1/SR-B1 transporter [37], followed by its delivery to the liver and its excretion via the bile. One lncRNA that amplifies LXR-mediated *Abca1* expression is lncRNA *MeXis* (Macrophage-expressed LXR-induced sequence) by guiding the transcriptional coactivator DDX17 to Abca1 enhancer regions [38]. In line, cholesterol efflux was increased in LXR-stimulated *MeXis*-expressing RAW cells, while lack of *MeXis* in bone marrow (BM) cells of WD-fed *Ldlr*^*−/−*^ mice accelerated atherosclerosis progression measured as lesion size and lipid content, compared with wild-type BM. LncRNA *ANRIL* (antisense non coding RNA in the INK4 locus) was reported to promote cholesterol efflux [39]. Mechanistically, ANRIL can function as scaffold lncRNA by recruiting DNA methyltransferase 1 (DNMT1) to the ADAM10 promoter, enhancing its methylation. The suppression of ADAM10 expression through overexpression of ANRIL in THP1 macrophage-derived foam cells and apolipoprotein E–deficient (*ApoE*^*−/−*^) mice showed increased cholesterol efflux and decreased lesion area. Of note, *ANRIL* has been extensively studied and reviewed elsewhere [40]. In contrast to promoting cholesterol efflux, lncRNA *GAS5* inhibited *Abca1* expression by binding the enhancer of zeste homologue 2 (EZH2), which in turn promotes triple methylation of lysine 27 (H3K27). Therefore, lncRNA *GAS5* reduced cholesterol efflux in THP-1 macrophage-derived foam cells and its knockdown decreased atherosclerosis progression in *ApoE*^*−/−*^ mice [41].

Next to lipid deposition, vascular inflammation initiates and drives atherosclerosis progression and is partly driven by activation of ECs through the NF-kB pathway [42]. Overexpression of lncRNA NEXN-AS1 (Nexilin F-actin binding protein antisense RNA 1) suppressed the TLR4/NF-kB pathway and thereby reduced endothelial activation and monocyte recruitment in human vascular endothelial cells (HVEC) [43]. Further, its overexpression inhibited proinflammatory pryoptosis-related biomarkers known to drive atherosclerosis (NLRP3, caspase-1, IL-1β, IL-18, GSDMD) [44]. Thereby, *NEXN-AS1* upregulates the expression of the *NEXN* gene by preventing chromatin condensation through binding the chromatin remodeler BAZ1A [43]. NEXN deficiency promoted atherosclerosis and plaque inflammation in WD-fed *ApoE*^*−/−*^ mice, while NEXN overexpression prevented these effects [43]. Additionally, the expression of both—*NEXN-AS1* and NEXN—is reduced in atherosclerotic arteries compared with healthy arteries in humans [43]. Further, atorvastatin significantly induced *NEXN-AS1* and NEXN expression, suggesting a new atheroprotective mechanism for statins non-lipid-lowering effects. lncRNA *MANTIS* (LncRNA n342419) also mediates vascular protection in *trans* via its interaction with SWI/SNF chromatin remodeling factor BRG1. This enables BRG1-promoter binding to angiogenic genes, such as SOX18 [45], and on the other hand, hinders BRG1 interaction with the promoter region of monocyte adhesion factor ICAM-1 [46]. As for *NEXN-AS1*, statins also increased *MANTIS* expression in human umbilical vein endothelial cells (HUVECs) and prevented reduced *MANTIS* expression in human artery endarterectomy compared with healthy vessels [46].

Although the majority of atherosclerotic plaque remain clinically silent, chronic inflammation and ongoing monocyte recruitment contribute to plaque growth and can feed into destabilization, thus, resulting in life-threatening acute events. One lncRNA found to be increased in unstable symptomatic compared with asymptomatic human atherosclerotic plaques was LncRNA-*CCL2* [47]*.* LncRNA-*CCL2* is upregulated in IL1-β-induced inflammatory HUVECs and regulates CCL2 mRNA levels in part through interaction with RNA-binding proteins in the nucleus, namely IGF2BP2 (insulin growth factor 2 binding protein 2) and HNRNPU [47]. The CCL2 gene encodes monocyte chemoattractant protein 1—a key mediator in inflammatory processes that facilitates monocyte recruitment and correlates with increased lncRNA*-CCL2* expression in symptomatic plaques. VSMCs can also contribute to inflammation, monocyte recruitment, and plaque destabilization via a phenotypic switch from a contractile state to synthetic macrophage-like cells [48]. This process was shown to be promoted by lncRNA NEAT1 (nuclear paraspeckle assembly transcript 1) [49]. *NEAT1* interacted with chromatin modifier WDR5 resulting in inhibited trimethylation at the promoters of genes encoding SM proteins.

Independent of the effects on circulating lipid levels or vessel wall inflammation was the observed accelerated atherosclerosis in high cholesterol diet (HCD)–fed *Ldlr*^*−/−*^ mice driven by the knockdown of lncRNA *SNHG12* [50]. LncRNA *SNHG12* (small nucleolar host gene-12) binds to DNA protein kinase (DNA-PK) in the vascular endothelium, which in turn facilitates binding of DNA-PKcs to Ku70/80 and the ability of DNA damage repair. Thus, *SNHG12* knockdown resulted in increased DNA damage and cellular senescence in vitro and in vivo, which exacerbated EC dysfunction and macrophage efferocytosis. Further, reduced SNHG12 expression in atherosclerotic specimen of pigs and human was inversely correlated with DNA damage and senescence.

## LncRNA-DNA Interactions in Atherosclerosis

LncRNA-DNA interactions are diverse, and several mechanisms for how lncRNAs recognize specific target sites have been proposed including polymerase tethering, hybridization, and DNA-binding protein–mediated recruitment [51] (Fig. 1). The binding of lncRNA to specific DNA regions can lead to the recruitment of proteins regulating epigenetic modulations (described above) as well as to positive or negative gene expression. In the context of atherosclerosis, lncRNA *FA2H-2* regulates autophagy and inflammation via binding the promoter of mixed lineage kinase domain-like protein (MLKL) gene. The subsequently suppressed MLKL expression increased autophagy flux and alleviated inflammatory damage induced by oxLDL in SMC and ECs [52]. Increased autophagy has been reported to be atheroprotective by preventing macrophages and smooth muscle cells to become foam cells and by alleviating inflammation [53–56], suggesting an atheroprotective role for lncRNA *FA2H-2*. Indeed, knockdown of lncRNA *FA2H-2* in HFD-fed *ApoE*^*−/−*^ mice showed increased MLKL expression, reduced autophagy flux, and enhanced inflammation and lesion area [52].

## LncRNA-RNA Interactions in Atherosclerosis

LncRNAs can be shuttled to the cytoplasm and modulate pre-mRNA splicing, mRNA stability, miRNA availability, and/or protein translation [57] (Fig. 1). One example for lncRNA-mRNA interaction is lncRNA *SMILR* (smooth muscle–induced lncRNA). *SMILR* did directly bind the mRNA of the mitotic protein CENPF (centromere protein F) and promoted the proliferation of VSMCs [58]. In agreement with VSMC proliferation conferring with plaque stability, increased *SMILR* levels were detected in unstable compared with stable human atherosclerotic plaques [59]. Thereby, *SMILR* may represent a valuable target to prevent adverse vascular remodeling after balloon angioplasty and vessel stenting.

The LXR-mediated lncRNA *CHROME* negatively regulates a number of miRNAs (miRNA miR-27b, miR-33a, miR-33b, miR-128) in human hepatocytes and macrophages [60]. One of the genes being post-transcriptional repressed by these miRNAs upon *CHROME* deficiency is *Abca1*. Thus, *CHROME* upregulates cholesterol efflux and HDL biogenesis, manifesting atheroprotective effects. Atherogenic outcomes via affecting cholesterol metabolism have been shown for lncRNAs *RP5-833A20.1* [61], *DAPK1-IT1* [62], and *GAS5* [63–66]. *RP5-833A20.1* induced hsa-miR-382-5p expression and in turn inhibited nuclear factor IA (NFIA) expression in macrophage-derived foam cells [61]. In vivo experiments using *ApoE*^*−/−*^ mice fed a high-fat/high-cholesterol diet confirmed the RP5-833A20.1/hsa-miR-382-5p/NFIA pathway and additional showed that overexpressing NFIA results in atheroprotective circulating lipoprotein changes, enhanced RCT, decreased circulating cytokine levels, and suppressed lesion formation. LncRNA *DAPK1-IT1* decreased miR-590-3p expression and led to increased LPL expression and reduced cholesterol efflux in THP-1 macrophage-derived foam cells [62]. Elevated *GAS5* (growth arrest-specific 5) levels are present in atherosclerotic plaque of human [66], rat [66], and mice [63] and promote inflammation, foam cell formation, and apoptosis as well as lipid disorders by interacting with miR-135a [63, 64] and miR-221 [65].

Another interesting lncRNA in atherosclerosis is *MALAT1* (metastasis-associated lung adenocarcinoma transcript 1). *MALAT1* regulates proliferation of ECs [67] and VSMCs [68] in vitro. Additional in vitro analyses revealed the interactions of *MALAT1* with miR-216-5p [69] or miR-22 [70] to promote autophagy and pryoptosis, respectively. A recently published study elucidated the in vivo role of *MALAT1* in atherosclerosis and demonstrated that MALAT1 exhibits anti-inflammatory properties in part by binding to miR-503 [71]. In detail, deficiency of lncRNA *MALAT1* in hematopoietic cells leads to enhanced atherosclerotic lesion formation and inflammation in HFD-fed *ApoE*^*−/−*^ mice [71]. The acceleration of lesion formation was driven by an increase in inflammatory BM cell number and enhanced adhesion to ECs in vitro and atherosclerotic vessel wall in vivo. Further, enhanced adhesion of BM cells was rescued by inhibition of miR-503. In line, *MALAT1* expression in human atherosclerotic plaque was downregulated in comparison to healthy vessel and, moreover, was decreased in symptomatic versus asymptomatic patients.

LncRNAs can also function as competitive endogenous RNA (ceRNA) regulating gene expression by sequestering miRNAs [72]. Although this concept has been questioned as computational analyses indicated the shortcoming of lncRNAs compared with the in excess expressed miRNA [73], several studies reported on lncRNA-miRNA interactions in the context of atherosclerosis. For example, lncRNA *MEG3* functions as sponge of miR-361-5p, regulating ABCA1 expression in VSMC [74] or the expression of ICAM-1 by sponging miR-147 [75]. Further, *CERNA1* (Competing Endogenous lncRNA 1 For MiR-4707-5p And MiR-4767, previously *LOC100129973*) inhibited apoptosis of VSMC and anti-inflammatory macrophages through increasing the apoptosis inhibitor API5 via sponging miR-4707-5p [76, 77]. Thereby, *CERNA1* overexpression in HFD-fed *ApoE*^*−/−*^ mice led to features of stable plaques, such as an increase in VSMCs and a decrease in MMP-2/9 activity, necrotic core area, and apoptotic cells. Another regulator of plaque vulnerability is lncRNA *MIAT* (Myocardial infarction associated transcript), which is upregulated in symptomatic human atherosclerotic specimen as well as in serum and plaques of HFD-fed *ApoE*^*−/−*^ mice [78]. Deficiency of *MIAT* in atherosclerotic *ApoE*^*−/−*^ mice improved efferocytosis, decreased apoptosis, and attenuated plaque growth. Mechanistically, *MIAT* acts as a sponge of miR-149-5p, subsequently inhibiting the mRNA degradation of the anti-phagocytic molecule CD47 in oxLDL-stimulated Raw264.7 cells. Moreover, lncRNA *TUG1* (taurine-up-regulated gene 1) acts as sponge for miR-148b in oxLDL-stimulated VSMC and HUVECs and regulated their proliferation and apoptosis via TUG1/miR-148b-promoted insulin growth-like factor 2 (IGF2) expression [79]. Another *TUG1* target is miR-133a [80]. Sponging miR-133a in oxLDL-simulated Raw264.7 upregulated fibroblast growth factor 1 (FGF1) expression, in turn leading to increased proliferation, inflammation, and inhibited apoptosis. In line with the in vitro data, *TUG1* knockdown in HFD-fed *ApoE*^*−/−*^ improved circulating lipid levels and inflammatory markers and reduced lesion size [80].

## Uncharacterized Interactions

The mechanisms for two recently described lncRNAs—*HOXA-AS2* and *PELATON*—implicated in atherosclerosis are yet to be defined. For example, transcriptomic profiling of HUVECs with and without the expression of lncRNA *HOXA-AS2* showed that *HOXA-AS2* mediates expression of inflammatory factors [81]. Further experiments confirmed that *HOXA-AS2* regulated endothelium inflammation by repressing NF-κB signaling. At the same time, NF-κB activity upregulated *HOXA-AS2*, which is likely the reason for the highly increased expression of HOXA-AS2 in human atherosclerotic plaques [81]. However, how exactly lncRNA HOXA-AS2 regulates inflammatory marker and especially the feedback loop with NF-κB is not yet known. LncRNA *PELATON* (Plaque enriched lncRNA in atherosclerotic and inflammatory bowel macrophage regulation) was found to be implicated in plaque instability and is enriched in unstable compared with stable atherosclerotic plaques [82]. With its high nuclear expression in monocytes and macrophages, it was shown to regulate phagocytosis, oxLDL uptake, and ROS production in differentiated primary human monocytes, in part due to changes in CD36 expression. However, the underlying mechanism is yet to be elucidated.

## LncRNAs as Biomarkers in Atherosclerosis

Finally, abundance of circulating lncRNAs or the occurrence of single nucleotide polymorphisms (SNPs) represents novel biomarkers for CVDs. Increased levels of lncRNAs *H19* and *LIPCAR* were found in plasma and serum samples in a Chinese population with atherosclerotic disease [83, 84]. Further, lncRNA *MIAT* were detected to be elevated in the blood of ischemic stroke patients [85]. LncRNA *SMILR* seems to play a role in plaque stability [58], and its plasma levels positively correlate with C-reactive protein [59].

SNPs in the lncRNA *MIAT* promoter correlated with acute myocardial infarction in a Chinese Han population [86], while a specific polymorphism in *MALAT1* (rs619586AG/GG) might be CVD protective [87].

## Conclusion

Collectively, cited studies in this review (Table 1) show that dysregulated lncRNAs are becoming a hallmark of atherosclerosis and play a role in several atherosclerosis processes as well as cell types. It has thus become clear that lncRNAs with their ability to interact with protein, DNA, and RNA can dynamically regulate the numerous functions of a variety of plastic cells and thereby impact atherosclerotic plaque growth, inflammation, and stability. However, understanding the complex regulation of lncRNAs in atherosclerosis is still in its infancy. Despite a number of lncRNAs being described in atherosclerosis, these mainly entail in vitro functions and less is known in respect to their roles in vivo*.* The scope of elucidating its functions in vivo is limited by the low conservation between species and impedes the investigation of the most interesting primate-specific lncRNAs in the widely used atherosclerotic mouse models. Further, there is also a big discrepancy between the number of discovered and functionally characterized lncRNAs. Notably, lncRNAs are not limited to the discussed binding properties in this review and likely have different roles depending on cell type and localization in subcellular compartments that may yet have to be defined. LncRNAs may also exhibit stage-specific roles, but, up to date, research has focused on atherosclerosis progression and information on lncRNAs in resolution of atherosclerosis is scant.

**Table 1:** LncRNAs in atherosclerosis

LncRNA		Binding partner	Function			Ref
			Human specimen	In vivo	In vitro	
*LASER*	Lipid Associated Single nucleotide polymorphism gEne Region	lncRNA-protein	Positively correlated with cholesterol levels in PBMC patients	N/A	Deficiency: ↓ cholesterol in HepG2 cells, further reduced with statin treatment	31
*LeXis*	Liver-expressed LXR-induced sequence	lncRNA-protein	N/A	Overexpression: C57BL/6 mice, AV *LeXis* ↓circulating and hepatic cholesterol content Deficiency: C57BL/6 mice, *LeXIS* ASO ↑ cholesterogenic gene expression ↑ serum levels	N/A	32
*MeXis*	Macrophage-expressed LXR-induced sequence	lncRNA-protein	N/A	Deficiency:*Ldlr*^*−/−*^ on WD *+ MeXis*^*−/−*^ bone marrow↓ Abca1 expression ↑ inflammatory gene expression ↑ lesion size ↑ CD68+ cells	Deficiency: Peritoneal macrophages (*MeXis*^*−/−*^ mice fed aWD) ↓ ABCA1 ↓ cholesterol efflux ↑ cholesterol accumulation	35
*ANRIL; CDKN2B-AS1*	Antisense non coding RNA in the INK4 locus	lncRNA-protein	↓ atherosclerotic plaque	Overexpression: *ApoE*^*−/−*^ on HFD, LV-induced OE ↑ Cholesterol efflux ↓ lesion size ↓ inflammation	Overexpression: THP-1 (oxLDL) ↑ cholesterol efflux ↓ lipid accumulation ↓ inflammation ↓ ADAM10	36, 37
*NEXN-AS1*	Nexilin F-actin binding protein antisense RNA 1	lncRNA-protein	↓ atherosclerotic plaques ↓ NEXN in CAD patients (blood)	Deficiency: *NEXN*^*±*^ */ApoE*^*−/−*^ on WD ↑ lesion area, macrophage abundance, expression of adhesion molecules ↑ inflammatory cytokines	Overexpression: HUVECs ↓ TLR4/NF-kB pathway ↓ inflammatory gene expression	40, 41
*MANTIS*		lncRNA-protein	↓ atherosclerotic plaque	Deficiency: Retinal injection of siRNA *MANTIS* ↑ ICAM-1	Deficiency: HUVECs ↓ angiogenic genes ↑ ICAM-1 ↑ monocyte adhesion ↑ apoptosis ↑ oxidative stress	42, 43
*LncRNA-CCL2*	C-C Motif Chemokine Ligand 2	lncRNA-protein	↑ unstable symptomatic atherosclerotic plaque	N/A	Deficiency: HUVEC (IL-1ß) ↓ CCL2	44
*NEAT1*	Nuclear paraspeckle assembly transcript 1	lncRNA-protein	N/A	Deficiency: *NEAT1*^*±*^ , Carotid Artery Ligation Injury ↓ VSMC proliferation and migration ↓ Neointima formation	Overexpression: ↑ VSMC proliferation and migration Deficiency:↓ VSMC proliferation and migration	46
*SNHG12*	Small nucleolar host gene-12	lncRNA-protein	Inverse correlation of SNHG12 expression with DNA damage and senescence markers in human atherosclerosis	Overexpression:*ApoE*^*−/−*^ mice on HCD ↓ lesion area ↓ lipid accumulation ↓ DNA damage (yH2AX) Deficiency: *Ldlr*^*−/−*^ on HCD, gapmeR-induced KO ↑ lesion area ↑ lipid accumulation ↑ plaque necrosis ↓ efferocytosis ↑ DNA damage (yH2AX)	Deficiency HUVEC ↑ yH2AX ↑ tail moment	47
*FA2H-2*	Fatty Acid 2-Hydroxylase 2	lncRNA-DNA	↓ atherosclerotic plaque	Deficiency: *ApoE*^*−/−*^ + LV-si-lncRNA-FA2H-2 on WD ↑ autophagy flux ↑ inflammatory response ↑ increased lesion area	Deficiency: ECs and SMCs (oxLDL) ↑ autophagy flux ↑ increased inflammatory response	49
*SMILR*	Smooth muscle–induced lncRNA enhances replication	lncRNA-RNA	↑ unstable atherosclerotic plaque ↑in plasma from patients with high plasma C-reactive protein	N/A	Deficiency: ↑ Proliferation of arterial and venous SMCs	55, 56
*CHROME*	Cholesterol Homeostasis Regulator of MiRNA Expression	lncRNA-RNA	↑ CAD (plasma), ↑ symptomatic versus asymptomatic atherosclerotic plaques	N/A	Deficiency: HepG2 cells, primary human hepatocytes, THP-1 ↓ ABCA1 protein expression ↓ cholesterol efflux to exogenous apoA-1	57
*RP5-833A20.1*		lncRNA-RNA	N/A	Overexpression: *ApoE*^*−/−*^ on HFD, LV-induced NFIA OE ↑ cholesterol efflux ↓ lesion size ↓ lipid accumulation	Overexpression: THP-1 (oxLDL) ↓ cholesterol efflux ↑ lipid accumulation ↑ miR-382-5p ↓ NFIA	58
*DAPK1-IT1*	DAPK1 Intronic Transcript 1	lncRNA-RNA	N/A	Overexpression: *ApoE−/−* on HFD, LV-induced LPL OE ↓ HDL-C, ↑ LDL-C ↑ circulating proinflammatory cytokines ↑ lesion size, ↑ lipid accumulation ↓ ABCA1	Overexpression: THP-1 (oxLDL) ↓ miR5903p ↑ LPL (same for def), ↑ total CH ↓ cholesterol efflux ↑ inflammatory cytokines Deficiency: THP-1 (oxLDL) ↑ miR5903p ↓ LPL	59
*GAS5*	Growth-arrest Specific 5	lncRNA-protein lncRNA-RNA	N/A	Overexpression: *ApoE*^*−/−*^ on HFD, LV-induced OE ↓ HDL-C, ↑ LDL-C ↓ reduced cholesterol efflux ↑ lesion size ↑ inflammation Deficiency: *ApoE*^*−/−*^ on HFD, sh-GAS5 ↑HDL-C, ↓ LDL-C ↑ reduced cholesterol efflux ↓ lesion size ↓ inflammation	Overexpression: THP-1 (oxLDL) ↓ cholesterol efflux ↑ lipid accumulation ↓ ABCA1 ↑ inflammatory markers ↑ MMP-2, MMP-9 ↑ EZH ↓ miR-135 ↓ miR-221 Deficiency: THP-1 (oxLDL) ↑ cholesterol efflux ↓ lipid accumulation ↑ABCA1 ↓ inflammatory markers ↓ MMP-	38, 60–63
*MALAT1*	Metastasis-associated lung adenocarcinoma transcript 1	lncRNA-RNA	↓ atherosclerotic plaque, correlates with symptoms of plaque instability	Deficiency: *ApoE*^*−/−*^ *Malat1*^*−/−*^ bone marrow cells on HFD ↑ adhesion to endothelial cells ↑ proinflammatory mediators ↑ lesion size ↑ miR-503	Deficiency: HUVECs (oxLDL) ↓ autophagy ↑ apoptosis ↑ miR-216a-5p EA.hy926 cells (high glucose) ↓ pryoptosis ↓ NLRP3 ↑ miR-22	64–68
*MEG3*	Maternally Expressed 3	lncRNA-RNA	N/A	Overexpression: *Ldlr*^*−/−*^ on HFD ↓ CD68+, CD3+, ICAM-1 ↑ collagen content	Overexpression: HMEC-1 ↓ cell viability, migration, tube formation ↑apoptosis suppress miR-147 Deficiency: VSMCs ↑ proliferation ↓ apoptosis ↓ ABCA1 suppress miR-361-5p	71, 72
*CERNA1*	Competing Endogenous lncRNA 1 For MiR-4707-5p And MiR-4767	lncRNA-RNA	N/A	Overexpression: ApoE−/− on HFD, LV-induced OE ↑ VSMCs ↓ IL-6 ↓ necrotic core area ↓ apoptosis	Overexpression: HUVEC (oxLDL) ↓ apoptosis ↓miR4707-5p Deficiency: HUVEC (oxLDL) ↑ apoptosis ↑miR4707-5p	73, 74
*MIAT*	Myocardial infarction associated transcript	lncRNA-RNA	↑ serum of patients with symptomatic atherosclerotic disease	Deficiency: *ApoE*^*−/−*^ on HFD, shRNA-induced KD ↓ lesion size ↓ necrotic core ↑ fibrous cap ↑ collagen ↑ efferocytosis	Deficiency: BMDM (oxLDL) ↑ phagocytosis Raw264.7 (oxLDL) ↑ miR-149-5p ↓ CD47	75
*TUG1*	Taurine upregulated gene 1	lncRNA-RNA	N/A	Deficiency: *ApoE*^*−/*−^ on HFD, siRNA-induced KD ↓ hyperlipidemia ↓ inflammatory response ↓ lesion size	Overexpression: RAW264.7 (oxLDL) ↑ cell growth ↓ apoptosis ↑ inflammation ↓ miR-133a Deficiency: VSMCs (oxLDL) ↓ proliferation ↑ apoptosis ↓ miR148b HUVECs (oxLDL) ↑ proliferation ↓ apoptosis ↓ miR148b	76, 77
*HOXA-AS2*	HOXA Cluster Antisense RNA 2	N/A	↑ atherosclerotic lesions	N/A	Deficiency: HUVECs ↑ NF-kB signaling, ↑ inflammatory response	78
*PELATON*	Plaque enriched lncRNA in atherosclerotic and inflammatory bowel macrophage regulation	N/A	↑ unstable atherosclerotic plaque	N/A	Deficiency: CD14+ hdPBMC ↓ phagocytosis ↓ oxLDL uptake ↓CD36	79

RNA-centered therapeutics in CVDs are already used in the clinics; for example, an RNA interfering agent to treat hypercholesterolemia [88]. Additional clinical trials are ongoing, but these do not yet involve lncRNAs [89, 90], as the relative lack of knowledge in the diverse and complex mechanisms of lncRNAs in atherosclerosis hinders its clinical translation. Nonetheless, the rapidly evolving advancements in genomic tools and increasing accomplishments to understand the biology of atherosclerotic lncRNAs hold promise for their clinical translation in the near future.

## Abbreviations

ABCA1: ATP-binding Cassette Transporter A1
ADAM: A Disintegrin and Metalloproteinase
ApoE: Apolipoprotein E
ASO: Antisense Oligonucleotides
BMDM: Bone Marrow-derived Macrophages
CCL2: C-C Motif Chemokine Ligand 2
CH: Cholesterol
EC: Endothelial Cell
EZH: Enhancer of Zeste Homologue
HCD: High-cholesterol Diet
HDLC: High-density Lipoprotein Cholesterol
HFD: High-fat Diet
HUVEC: Human Umbilical Vein Endothelial Cells
ICAM-1: Intercellular Adhesion Molecule 1
LDL-C: Low-density Lipoprotein Cholesterol
LDLR: LDL Receptor
LPL: Lipoprotein Lipase
LV: Lentivirus
MMP: MatrixMetallopeptidase
NFIA: Nuclear Factor IA
NLRP3 NOD: LRR- and Pyrin domain-containing protein 3
PBMC: Peripheral Blood Mononuclear Cell
OE: Overexpression
oxLDL: oxidized LDL
siRNA: small interferenceRNA
THP-1: human acute monocytic leukemia cell line
VSMC: VascularSmooth Muscle Cells
WD: Western Diet
